# NiftySim: A GPU-based nonlinear finite element package for simulation of soft tissue biomechanics

**DOI:** 10.1007/s11548-014-1118-5

**Published:** 2014-09-21

**Authors:** Stian F. Johnsen, Zeike A. Taylor, Matthew J. Clarkson, John Hipwell, Marc Modat, Bjoern Eiben, Lianghao Han, Yipeng Hu, Thomy Mertzanidou, David J. Hawkes, Sebastien Ourselin

**Affiliations:** 1 Centre for Medical Image Computing, University College London, London, UK; 2 Department of Mechanical Engineering, CISTIB Centre for Computational Imaging and Simulation Technologies in Biomedicine, Insigneo Institute for in silico Medicine, The University of Sheffield, Sheffield, UK

**Keywords:** FEM, Total Lagrangian explicit dynamics, GPU, Software engineering, Soft tissue biomechanics

## Abstract

**Purpose:**

*NiftySim*, an open-source finite element toolkit, has been designed to allow incorporation of high-performance soft tissue simulation capabilities into biomedical applications. The toolkit provides the option of execution on fast graphics processing unit (GPU) hardware, numerous constitutive models and solid-element options, membrane and shell elements, and contact modelling facilities, in a simple to use library.

**Methods:**

The toolkit is founded on the total Lagrangian explicit dynamics (TLEDs) algorithm, which has been shown to be efficient and accurate for simulation of soft tissues. The base code is written in C$++$, and GPU execution is achieved using the nVidia CUDA framework. In most cases, interaction with the underlying solvers can be achieved through a single Simulator class, which may be embedded directly in third-party applications such as, surgical guidance systems. Advanced capabilities such as contact modelling and nonlinear constitutive models are also provided, as are more experimental technologies like reduced order modelling. A consistent description of the underlying solution algorithm, its implementation with a focus on GPU execution, and examples of the toolkit’s usage in biomedical applications are provided.

**Results:**

Efficient mapping of the TLED algorithm to parallel hardware results in very high computational performance, far exceeding that available in commercial packages.

**Conclusion:**

The *NiftySim* toolkit provides high-performance soft tissue simulation capabilities using GPU technology for biomechanical simulation research applications in medical image computing, surgical simulation, and surgical guidance applications.

## Introduction

In this paper, we describe the development and features of the open-source finite element (FE) toolkit, *NiftySim*. The toolkit’s key feature is its use of graphics processing unit (GPU)-based execution, which allows it to outperform equivalent central processing unit (CPU)-based implementations by more than an order of magnitude, and commercial packages by significantly more again [9, 29]. While the solver may be used for the analysis of any solid materials, it has been designed and optimised for simulation of soft tissues. The motivation for its development is the growing need for robust soft tissue modelling capabilities in medical imaging and surgical simulation applications, and in particular, in time-critical applications. The latter include, for example, interactive simulation systems where real-time computation is required [5, 19, 24], and intra-operative image registration and image guidance systems [2, 3, 7] for which rapid, if not real-time, computation is necessary.

*NiftySim* was developed around the total Lagrangian explicit dynamic (TLED) FE algorithm, first identified as a potentially efficient approach for soft tissue simulation by Miller et al. [21] (but, see also [24]). An important feature of the presented algorithm is that it correctly accommodates geometric and constitutive nonlinearities, both of which are essential for this application; soft tissues generally can tolerate large deformations, and their stress–strain response is seldom linear [11]. The efficiency of the algorithm derives from two aspects: (1) the total Lagrangian framework allows shape function derivatives to be precomputed and stored, rather than re-computed at each time step and (2) the low stiffness of biological tissues means the critical time steps for explicit integration, normally a very restrictive constraint, are relatively large. Since explicit methods involve comparatively inexpensive computations in each time step, the latter feature can lead to very low overall computation times.

An additional virtue of explicit methods that is central to *NiftySim* ’s development is their amenability to parallel execution. Whereas the main computational task in implicit methods is solution of a large linear system (several times per time step for nonlinear problems), computations in explicit solution procedures are executed on an element- and node-wise basis. The mapping to parallel hardware is thus direct and efficient. This fact was exploited in our earlier work [25, 26] to produce a GPU-based solver using OpenGL and the Cg graphics language. The introduction of the general-purpose CUDA API [22] allowed a more flexible and efficient implementation to be proposed subsequently, as described in [27, 28]. In separate work, we also described the incorporation of the technology in the SOFA framework [4]. The underlying technology in *NiftySim* builds on the approach described in [28], in particular.

*NiftySim* also includes a number of features that go beyond the solid-element-based TLED algorithm, the most important of which are: (1) membrane and shell formulations compatible with TLED’s explicit time integration (described in [1] and [8], respectively) that can be used on their own or in conjunction with solid-element-based meshes, (2) specialised contact models for the efficient simulation of interactions between deformable geometry and simple, analytically describable surfaces, (3) a general-purpose mesh-based contact model with a collision response formulation derived from the work of Heinstein et al. [10, 15]. The latter can simulate contacts between multiple deformable bodies, self-collisions, and contacts between deformable geometry and rigid surfaces.

With its lightweight, yet consistent and flexible implementation of the TLED algorithm, written in C$++$ and CUDA, *NiftySim* is primarily aimed at researchers developing algorithms in the area of medical image analysis, surgical image guidance, and surgical simulation, requiring a fast FE backend for the simulation of soft tissue mechanics. It is mainly geared towards an algorithmic generation of simulation descriptions and post-processing of results with custom researcher-written code. Therefore, our goal is not to compete with end-to-end toolkits like SOFA^1^ that provide their own tools for graphical simulation definition and interaction, or general-purpose finite element analysis suites like Abaqus FEA.^2^ Further, unlike the common commercial packages, which must be accessed via the command line, *NiftySim* can be used as a back-end library in C$++$ applications, thus allowing for the direct exchange of data with client code. To aid the integration of *NiftySim* in such specialised applications, it sports the following features: It has been tested on various versions of Linux, Mac OS and Windows. A command line application capable of executing complete simulations and that can be used in conjunction with scripting languages or for prototyping simulations is included. Various features simplifying its use as a library are also available, such as a wrapper simulator class, which encapsulates all of the simulation technology and allows it to be easily embedded in other libraries and applications, and full support for CMake’s^3^ *config mode*.

In the remainder of the paper, we give a brief introduction to *NiftySim* ’s usage (see section “NiftySim usage”). Full details of the continuum formulation and solution algorithms can be found in our earlier publications [26, 28, 30]; however, a summary of the core algorithm is provided (see section “The TLED algorithm”), followed by a description of the main classes and their implementation in section “Implementation using C$++$/CUDA”, outline some example applications taken from published research that employed *NiftySim* (see section “Research applications of NiftySim”), and conclude with a brief discussion (see section “Discussion and conclusions”). A description of the constitutive models currently available is provided in the “Appendix”.

The toolkit is available for download from SourceForge^4^ and subject only to the terms of a liberal BSD-style licence.

## NiftySim usage

This section gives a brief overview of *NiftySim* ’s usage by means of two simple examples. For a more comprehensive description, the reader is referred to *NiftySim* ’s PDF user manual that ships with the source code.

**Fig. 1:**
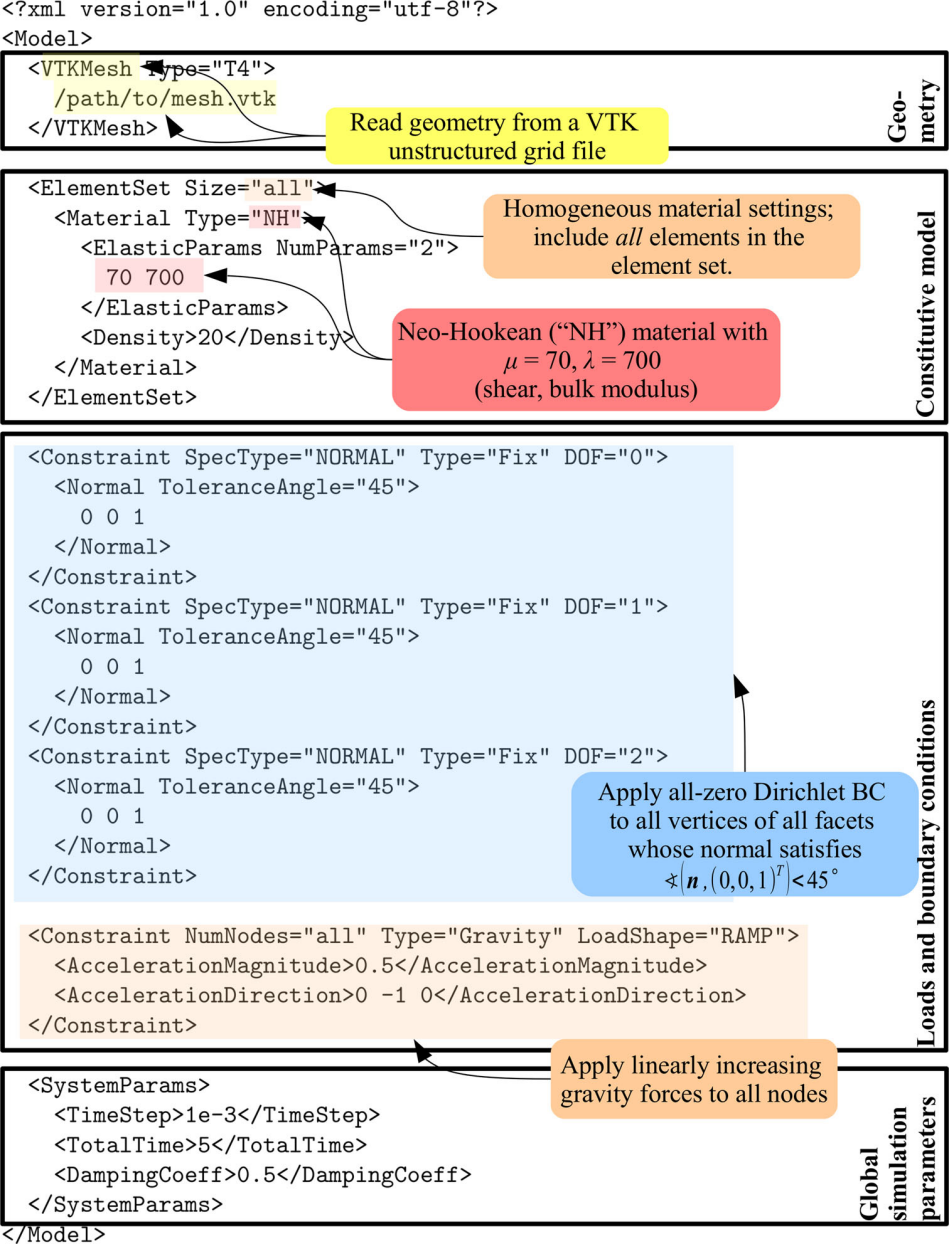
An annotated *NiftySim* simulation model

*NiftySim* can be used as a stand-alone application and as a library. However, it is used, the quickest and most flexible way to create a simulation is to describe it using *XML*. Figure 1 contains such a description, a *model*, for a simple *NiftySim* simulation comprising all parts found in a realistic simulation. The figure also introduces concepts such as *system parameters* and *element set* that will reappear later in the text.

**Fig. 2:**
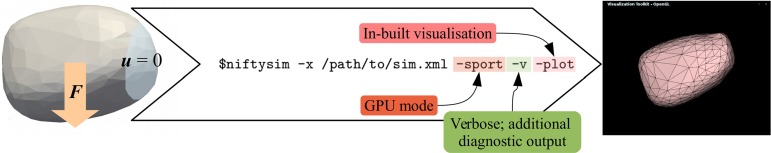
Execution of the simulation defined in Fig. 1 via *NiftySim* ’s stand-alone executable. *Left* Input geometry with constraints. *Right* Visual output of final configuration via *NiftySim* ’s in-built visualisation facilities. *Centre* Corresponding annotated command line

Figure 2 contains the first example showing the usage of *NiftySim* ’s stand-alone executable. It also contains an illustration of the constraints of the example model of Fig. 1.

**Fig. 3:**
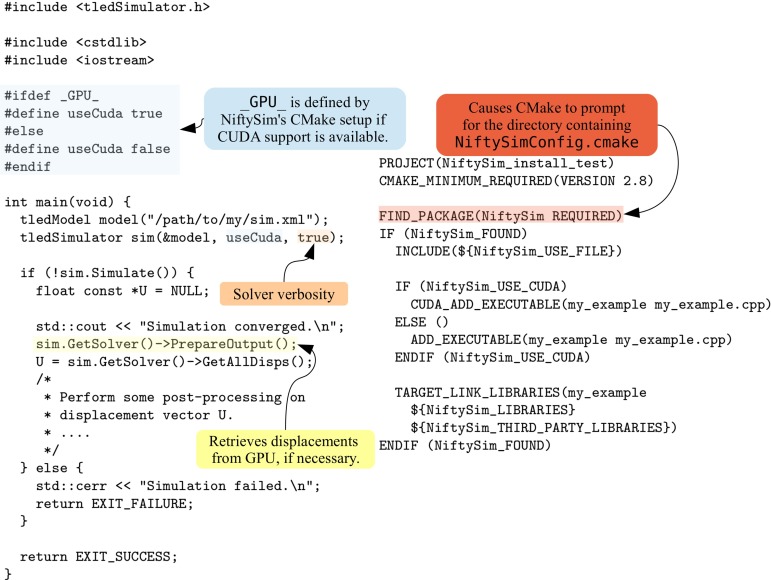
*Left* A simple C$++$ application that uses displacements computed with *NiftySim*. *Right* The corresponding CMakeLists.txt that takes care of the inclusion of the required *NiftySim* resources

Assuming the displacement field generated by the simulation is to be used with custom C$++$ code, e.g.—as in many of the research examples presented in section “Research applications of NiftySim”—to warp an image, using *NiftySim* as a library in a C$++$ code is the most advantageous. The simple C$++$ application in Fig. 3, consisting of a single compilation unit, my_example.cpp, containing only a main function, and a CMakeLists.txt for the build configuration, accomplishes the task of running *any* *NiftySim* simulation contained in the file residing at the hardcoded location /path/to/my/sim.xml.

## The TLED algorithm

### The basic TLED algorithm

At its core, TLED as described by Miller et al. [21] is an algorithm for the treatment of large deformation dynamic problems defined on a domain $\varOmega \subset \mathbb {R}^3$ for a time period $[0, T]$ given by an equilibrium equation of the form

1 $$\begin{aligned} \underbrace{\rho \varvec{\ddot{u}}(\varvec{x}, t)}_{\text {inertia}} + \underbrace{\nabla \cdot \varvec{\sigma }\left( \varvec{u}(\varvec{x}, t)\right) }_{\text {internal forces}} {=} \underbrace{\varvec{f}(\varvec{x}, t)}_{\text {body forces}},\quad \varvec{x} \in \varOmega ,\;t \in [0, T] \end{aligned}$$

where $\rho$ is the material’s mass density, $\varvec{\sigma }$ denotes the Cauchy stress in the simulated body, and $\varvec{u}$ is the displacement field and $\varvec{\ddot{u}}$ the corresponding acceleration.

The Dirichlet and Neumann BCs corresponding to Eq. (1) are given by:

2 $$\begin{aligned} \varvec{u}(\varvec{x},t)&= \varvec{u}_{\text {constraint}}^t,\quad \varvec{x} \in \varGamma _u\nonumber \\ f(\varvec{x},t)&= \varvec{f}_{\text {constraint}}^t,\quad \varvec{x} \in \varGamma _f \end{aligned}$$

Performing the usual substitution of a piece-wise linear approximation for the displacement field $\varvec{u}$ and casting into the weak form via Galerkin weighting, the semi-discretised form of Eq. (1) becomes

3 $$\begin{aligned} \varvec{M} \ddot{\varvec{U}} + \varvec{D} \dot{\varvec{U}} + \varvec{R}^\mathrm{int} \left( \varvec{U} \right) = \varvec{R}^\mathrm{ext} \end{aligned}$$

where $\varvec{M}$ is the *lumped*, i.e. diagonal, mass matrix and $\varvec{D}$ is a diagonal damping matrix, introduced for the numerical stability of the time integration. In TLED the latter is linked to the mass matrix via a damping coefficient $\alpha _D$: $D = \alpha _D M$. $\varvec{R}^\mathrm{ext}$ are the discretised external loads, i.e. body forces and Neumann BCs.

The internal force term, $\varvec{R}^\mathrm{int}$ in Eq. (3), is given by4where  is the assembly operator performing the accumulation of the element internal forces, $\varvec{f}^{(e)}$, that are in turn given by

5 $$\begin{aligned} \varvec{f}^{(e)} = \int _{V^e} \partial _{\varvec{X}} \varvec{h} \varvec{S} \varvec{F}^\mathrm{T} \, \mathrm{d}V^e \end{aligned}$$

where $\varvec{\partial _{X} h}$ are the derivatives of the shape functions $\varvec{h}$ with respect to the reference configuration coordinates $\varvec{X}$, $\varvec{S}$ is the second Piola–Kirchhoff stress computed with one of the constitutive models given in the section “Constitutive models” in Appendix, and $V^e$ denotes the volume of element $e$. The deformation gradient $\varvec{F}$ is defined as

6 $$\begin{aligned} \varvec{F} = \frac{\partial \varvec{x}}{\partial \varvec{X}} = \varvec{I} + \sum _i^{N_{\text {nodes/element}}} \varvec{U}_i\cdot \partial _{\varvec{X}} h_i \end{aligned}$$

with $\varvec{x}$ being the current and $\varvec{X}$ the initial position of a material point, and $\varvec{I}$ denoting the $3\times 3$ identity matrix

Use of the total Lagrangian evaluation of stresses means the shape function derivatives $\partial _{\varvec{X}} \varvec{h}$ only need to be computed once.

TLED employs one-point quadrature on the spatial domain, meaning the numerical approximation of $\varvec{f}^{(e)}$ for the internal forces are evaluated only at the initial configuration centre of the corresponding element. One of the following formulas is used, depending on the element type that is employed in the discretisation of the problem:

*Linear 8-node reduced-integration hexahedron*  This element employs trilinear shape functions, and the formula for its internal forces is given by

7 $$\begin{aligned} \varvec{f}^{(e)} = 8 \det (\varvec{J}) \partial \varvec{h} \varvec{S} \varvec{F}^\mathrm{T}, \end{aligned}$$

where $\varvec{J}$ is the element Jacobian matrix. A well known deficiency of the element is its susceptibility to spurious zero-energy modes—so-called hourglass modes. These are controlled using the efficient method proposed by Joldes et al. [16].

*Linear 4-node tetrahedron*  This element employs linear shape functions. The formula (5) for element nodal forces is then

8 $$\begin{aligned} \varvec{f}^{(e)} = V^e \partial \varvec{h} \varvec{S} \varvec{F}^\mathrm{T}. \end{aligned}$$

It should be noted that this element is generally overly stiff, especially for nearly incompressible materials like soft tissues [14]. The nodal-averaged pressure tetrahedron, below, is preferable in most cases.

*Nodal-averaged pressure 4-node tetrahedron* Developed to alleviate the volumetric locking problems that plague the standard tetrahedron, this element employs the same shape functions and nodal forces formula (Eq. 8). The stress $\check{\varvec{S}}$, however, is computed using a modified deformation gradient whose volumetric component has been averaged over adjacent nodes—see [17]. The performance of this formulation is generally superior to that of the standard tetrahedron.

The other major reason for the algorithm’s efficiency is its treatment of the time ordinary differential equation (ODE). Two distinct explicit ODE solvers are implemented in *NiftySim*:

*Explicit Central-Difference Method (CDM)*: With this method solving for the next time-step displacements, $\varvec{U}_{n+1}$, at a given time step $n$, is achieved by substituting the following approximations for the velocity, $\varvec{\dot{U}}$, and the acceleration, $\varvec{\ddot{U}}$, into Eq. (3):

9 $$\begin{aligned} \varvec{\ddot{U}}_n&\approx \frac{1}{\varDelta t^2}\left( \varvec{U}_{n+1} - 2\varvec{U}_n + \varvec{U}_{n-1}\right) \nonumber \\ \varvec{\dot{U}}_n&\approx \frac{1}{2\varDelta t}\left( \varvec{U}_{n+1} - \varvec{U}_{n-1}\right) \end{aligned}$$

with $\varDelta t$ denoting the time step size. Solving for the next time-step displacements yields

10 $$\begin{aligned} \varvec{U}_{n+1} = \varvec{A}\left( \varvec{R}^\mathrm{ext} - \varvec{R}^\mathrm{int}\right) + \varvec{B}\varvec{U}_n + \varvec{C}\varvec{U}_{n-1} \end{aligned}$$

where the following coefficient diagonal matrices have been introduced:

11 $$\begin{aligned} A_{ii}&= 1\Big /\left( \frac{D_{ii}}{2\varDelta t} + \frac{M_{ii}}{\varDelta t^2}\right) \nonumber \\ B_{ii}&= \frac{2M_{ii}}{\varDelta t^2}\Big /\left( \frac{D_{ii}}{2\varDelta t} + \frac{M_{ii}}{\varDelta t^2}\right) \nonumber \\ C_{ii}&= \left( \frac{D_{ii}}{2\varDelta t} - \frac{M_{ii}}{\varDelta t^2}\right) {\Big /}\left( \frac{D_{ii}}{2\varDelta t} {+} \frac{M_{ii}}{\varDelta t^2}\right) ,i {=} 1,\ldots , N_{\text {nodes}}\nonumber \\ \end{aligned}$$

These coefficients are time-invariant and can be precomputed.

*Explicit Newmark Method (EDM)* This method introduces a numerical acceleration and velocity. It is summarised by the following formulas:

12 $$\begin{aligned}&\varvec{\ddot{U}}_n {=} \frac{1}{1 {+} \alpha _D\varDelta t/2}\left( M^{-1}\varvec{R}^\mathrm{eff} {-} \alpha _D \varvec{\dot{U}}_{n-1} - \frac{\alpha _D\varDelta t}{2}\varvec{\ddot{U}}_{n{-}1}\right) \nonumber \\&\varvec{\dot{U}}_n = \varvec{\dot{U}}_{n-1} + \frac{\varDelta t}{2}\left( \varvec{\ddot{U}}_{n} + \varvec{\ddot{U}}_{n-1}\right) \\&\varvec{U}_{n+1} = \varvec{U}_{n} + \varDelta t\varvec{\dot{U}}_n + \frac{\varDelta t^2}{2}\varvec{\ddot{U}}_n \nonumber \end{aligned}$$

As with CDM, coefficient diagonal matrices can be precomputed to accelerate the process.

Dirichlet BCs are incorporated at the end of a time step via a simple substitution of fixed values for the components of the displacement vector $\varvec{U}$ that are subject to such constraints.

### Acceleration of TLED by means of reduced order modelling

*NiftySim* also provides reduced order modelling (ROM) capabilities, the mathematical underpinnings of which are explained in detail in [29, 30]. The key idea is to project the full displacement field, defined by the usual vector of nodal values $\mathbf {U} \in \mathbb {R}^{3N_{\text {nodes}}}$, onto a lower dimensional basis $\varvec{\Phi } \in \mathbb {R}^{3N_{\text {nodes}} \times M}$ as follows:

13 $$\begin{aligned} \mathbf {U} = \varvec{\Phi } \mathbf {P},\quad \dot{\mathbf {U}} = \varvec{\Phi } \dot{\mathbf {P}},\quad \ddot{\mathbf {U}} = \varvec{\Phi } \ddot{\mathbf {P}}, \end{aligned}$$

where the latter two relations follow from the time-independence of $\varvec{\Phi }, \mathbf {P} \in \mathbb {R}^{M}$ is a vector of so-called generalised displacements, and $M \ll N_{\text {nodes}}$. The reduced basis $\varvec{\Phi }$ is computed using proper orthogonal decomposition of a training set of full model solutions. Each of the $M$ columns of $\varvec{\Phi }$ represents a mode of deformation of the structure and, as shown in (13), the full order displacements $\mathbf {U}$ are approximated by a linear combination of these modes, weighted by the generalised displacements $\mathbf {P}$.

Substitution of (13) into (3) and pre-multiplying by $\varvec{\Phi }^T$ yields

14 $$\begin{aligned} \hat{\mathbf {M}} \ddot{\mathbf {P}} + \alpha _D \hat{\mathbf {M}} \dot{\mathbf {P}} = \hat{\mathbf {R}}^{\text {eff}} \end{aligned}$$

where $\mathbf {D} = \alpha _D \mathbf {M}$ has been used, and $\hat{\mathbf {M}} \in \mathbb {R}^{M \times M}$ and $\hat{\mathbf {R}}^{\text {eff}} \in \mathbb {R}^{M}$ are the reduced mass matrix and effective nodal load vector, respectively, given by:

15 $$\begin{aligned} \begin{aligned}&\hat{\mathbf {M}} = \varvec{\Phi }^T \mathbf {M} \varvec{\Phi } \\&\hat{\mathbf {R}}^{\text {eff}} = \varvec{\Phi }^T \mathbf {R}^{\text {eff}} \end{aligned} \end{aligned}$$

with $\mathbf {R}^{\text {eff}} = \mathbf {R}^{\text {ext}} - \mathbf {R}^{\text {int}}$. Integrating the reduced equilibrium Eq. (14) using CDM results in a new incremental displacement update formula:

16 $$\begin{aligned} \mathbf {U}^{n+1} = \gamma _1 \varvec{\Phi } \hat{\mathbf {M}}^{-1} \varvec{\Phi }^T \mathbf {R}^{\text {eff}} + \gamma _2 \mathbf {U}_n + \gamma _3 \mathbf {U}_{n-1}, \end{aligned}$$

where $$\gamma _1 = 2 \varDelta t^2 / (\alpha _D \varDelta t + 2)$$, $$\gamma _2 = 4 / (\alpha _D \varDelta t + 2)$$ and $$\gamma _3 = 1 - \gamma _2$$.

The benefit conferred by this process is a substantial enlargement of the critical time step $\varDelta t_{\text {cr}}$, meaning many fewer time steps are required for a given simulation. In ref. [30], it was shown that speed improvements of around an order of magnitude are feasible, with an error below 5 % compared with full model solutions.

### Incorporation of membranes and shells in TLED

The membrane element implemented in *NiftySim* is based on ref. [1]. It is an iso-parametric triangle element in which the strain is computed via the usual reference triangle

17 $$\begin{aligned} T_{\text {ref}} = \{(0, 0), (1, 0), (0, 1)\} \end{aligned}$$

from the Jacobian matrices of the mappings from the reference to the current and the initial configurations

18 $$\begin{aligned} \begin{aligned}&\varvec{F_0} = \frac{\mathrm{d}\varvec{X}}{\mathrm{d}\varvec{\xi }},\quad \varvec{F_n} = \frac{\mathrm{d}\varvec{x}}{\mathrm{d}\varvec{\xi }}\\&\varvec{C_0} = \varvec{F_0^T}\varvec{F_0},\quad \varvec{C_n} = \varvec{F_n^T}\varvec{F_n} \end{aligned} \end{aligned}$$

The only available constitutive model for this element as of *NiftySim* version 2.3 is incompressible neo-Hookean, whose SPK stress is given by

19 $$\begin{aligned} \varvec{S}_{\varvec{\xi }} = \mu \left( \varvec{C_0}^{-1} - \frac{ II _{C_0}}{ II _{C_n}}\varvec{C_n}^{-1}\right) \end{aligned}$$

where $\mu$ is the shear modulus, and the strain invariant $II _{\varvec{C}} = \det (\varvec{C})$ was introduced.

The membrane internal forces are then given by

20 $$\begin{aligned} \varvec{f}^{(e)} = A^{e}H^{e} (\varvec{F_n}\varvec{S_{\xi }}) : \varvec{\partial _{\varvec{\xi }} h} \end{aligned}$$

with $A^e$ and $H^{e}$ denoting the initial element area and thickness, respectively, and the subscript $\varvec{\xi }$ indicating quantities evaluated on the reference triangle.

**Fig. 4:**
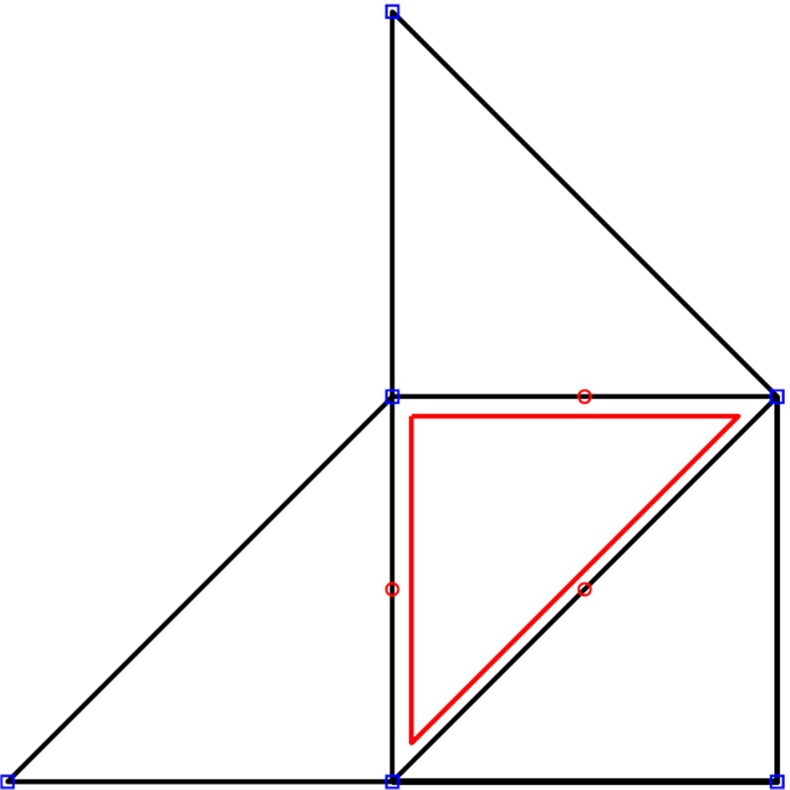
The 4-triangle patch underlying the calculations with the EBST1 shell element. The *central triangle* and its sampling points are highlighted in *red*. The *blue boxes* show the location of the six quadratic shape functions

The shell element supported by *NiftySim* is the rotation-free EBST1 described in [8]. Computations with this element are based on quadratic shape functions defined on patches consisting of four triangles (Fig. 4) with deformation and curvature functions being sampled at the midpoints of the edges of patches’ central triangle and subsequently averaged. With this shell element, the curvature giving rise to its bending stiffness is computed from standard nodal displacements; therefore, there is no need for modifications to the time-ODE solver algorithms employed with TLED.

The standard neo-Hookean model is currently the only available constitutive model for the membrane component; the bending moments are computed from the linear expression:

21 $$\begin{aligned} \varvec{m} = \frac{E{H^e}^3}{12(1 - \nu ^2)}\left( \begin{array}{c@{\quad }c@{\quad }c} 1 &{} \nu &{} 0\\ \nu &{} 1 &{} 0\\ 0 &{} 0 &{} (1 - \nu )/2 \end{array}\right) \varvec{\kappa } \end{aligned}$$

with $E$ and $\nu$ denoting Young’s modulus and the Poisson ratio, $\varvec{\kappa }$ being the curvature. The constitutive models for the membrane and bending component were taken from [23].

### Contact modelling

All contact modelling in *NiftySim* is based on prediction–correction, i.e. the basic TLED algorithm is used compute a prediction for the next time-step displacement, which is then used to search for potential contacts. If contacts are found, corrections must be computed. These can either be displacement corrections, directly applied to the displacement value of offending nodes, or collision response forces which are incorporated in the effective load vector, $\varvec{R}^\mathrm{eff}$.

In the simpler of the two contact modelling algorithms implemented in *NiftySim*, the penetration of deformable-geometry nodes into the *master* surface is found by evaluating an analytical expression. In this contact modelling context, the deformable geometry surface is referred to as the slave surface.

The master-surface description must allow for the evaluation of a *gap function*, denoted with $g$, whose value represents the signed distance to the closest point on the master surface, and if negative, indicates that the slave node has penetrated the master surface. This also implies that there must be a means of computing the surface normal, $\varvec{n_m}$, at every point on the master surface. The latter two quantities, $g$ and $\varvec{n_m}$, can then be used to compute a displacement correction, $\varvec{\varDelta u}$:

22 $$\begin{aligned} \varvec{\varDelta u} = -g\varvec{n_m} \end{aligned}$$

The pipeline for modelling mesh–mesh contacts implemented in *NiftySim* detects collisions of slave-surface nodes and the interior of master-surface facets and intersection of slave and master surface edges with bounding volume hierarchies (BVHs). The contact search algorithm returns a projection of slave nodes onto the master surface, here denoted with $(\xi ,\,\eta )$, as well as the corresponding gap function value, and in the case of edge–edge intersections, the signed shortest distance between the two edges at the end of the time step along with the corresponding edge parameters, labelled $r,\ q$. The formulas for the forces applied in response to collisions are derived from the explicit Lagrange-multiplier method of Heinstein et al. [10]. In the case of contacts between deformable bodies, the node-facet collision response forces are given by

23 $$\begin{aligned} \begin{aligned}&\varvec{f_s} = -\varvec{n_m}(\xi , \eta )\beta _s\frac{m_s g}{\varDelta t^2}\\&(\varvec{f_m})_i = \varvec{n_m}(\xi , \eta )\beta _m\frac{(m_m)_i g\gamma _i(\xi , \eta )}{\varDelta t^2},\\&\qquad \quad \qquad i \in \{\text {master-facet vertices}\}\\&\beta _s = \frac{m_m}{m_s + m_m},\quad \beta _m = 1 - \beta _s = \frac{m_s}{m_s + m_m} \end{aligned} \end{aligned}$$

where $\varvec{f_s}$ and $\varvec{f_m}$ denote the forces applied to the slave node and the master facet, respectively, $m_m$ is the mass associated with a virtual node placed at the point on the master facet that is closest to the slave node, $m_s$ denotes the mass of the slave node.

24 $$\begin{aligned} \gamma _i(\xi , \eta ) := \frac{h_i(\xi , \eta )}{\sum _j^{N_{\text {nodes/facet}}} h_j(\xi , \eta )^2},\quad i\in {1,\ldots ,N_{\text {nodes/facet}}} \end{aligned}$$

Is a coefficient computed from shape-function values, used to distribute forces among the vertices of the master facets, and is derived in [18].

The corresponding formulas for edge–edge collisions read

25 $$\begin{aligned}&(\varvec{f}_{s})_i = -\varvec{n}(r)\beta _s\frac{(m_s)_i\gamma (q)_ig}{\varDelta t^2}, \quad i\in \{0,1\}\nonumber \\&(\varvec{f}_{m})_i = \varvec{n}(r)\beta _m\frac{(m_m)_i\gamma (r)_ig}{\varDelta t^2}, \quad i\in \{0,1\} \end{aligned}$$

These collision response forces can be directly incorporated in the effective loads and used to update the displacement vector through a second evaluation of the CDM/EDM formulas (10)/(11).

### Implementation overview

The processing of a simulation with *NiftySim* consists of three main stages. The first stage deals with the parsing of the simulation XML description and the loading of the simulation geometry. In the precomputation step, the spatial derivatives of the shape functions, the node masses, and constraint and contact modelling-related data are computed. In typical usage scenarios, the precomputation happens absolutely transparently to the user in the *simulator* class’s constructor.

**Fig. 5:**
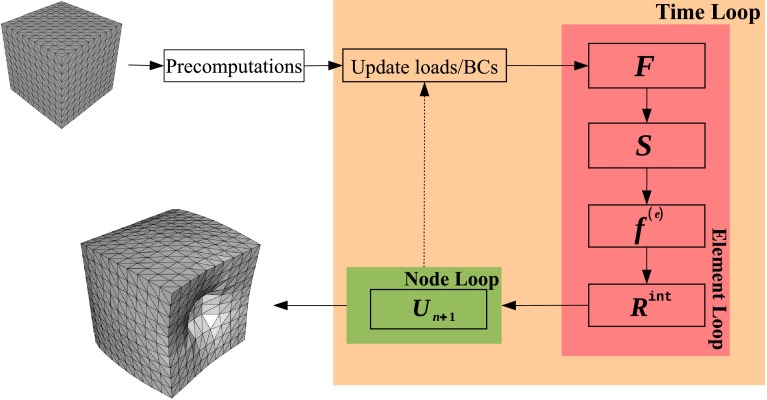
Flowchart representation of *NiftySim* ’s simulation pipeline

When the precomputation is finished, the simulator initialises the solution variables and constraints and enters the main loop. The main loop iterates over the simulation time steps. In every time step, at the very least, the internal forces of the structure and, based on these forces, displacements must be updated. Figure 5 shows a graphic representation of *NiftySim* ’s workflow.

In a minimal, sequential TLED implementation, Eq. (4) can be evaluated in one loop over all elements, computing in every element its deformation gradient, strains, stresses and from that internal forces, and accumulating the per-element internal forces in a global internal-force vector. With this done, the effective loads can be computed by subtracting the internal forces from the applied external loads. A second loop is then invoked, iterating over the nodes in the mesh and updating their displacements based on Eq. (10). Thanks to the lumping of the mass matrix, this last step can be done for each node individually. Parallel implementations require a more complex memory layout to efficiently avoid race conditions on the internal-force accumulation buffer. The basic pattern of two main loops, one over all elements and one over all nodes, remains the same, though. A more detailed description of the strategies employed in *NiftySim* ’s parallel solvers is given in section “The solver classes”.

## Implementation using C$++$/CUDA

This section introduces the most important modules and concepts of *NiftySim* ’s TLED implementation. A more complete list and technical description of *NiftySim* ’s modules can be found in the source code’s *Doxygen* 5 documentation.

### Coding guidelines and naming conventions

*NiftySim* follows VTK^6^ naming conventions, where class names have a “tled” prefix and are camel-cased, e.g. tledExampleNiftySimClass. Member names are also camel-cased and start with a capital letter. Names of functions normally begin with an appropriate verb.

Function signatures were until recently also based on VTK’s style with no function arguments and member functions having const modifiers. Motivated by the addition of CPU parallel solvers and the potential race conditions it entails, a move towards a style more similar to that of the Insight Segmentation and Registration Toolkit^7^ has been undertaken, where certain member functions such as getters have const modifiers, as do all read-only function arguments.

The CUDA portion of *NiftySim* was designed to be as far as possible backward compatible; the use of complex classes in CUDA device code is therefore avoided. Instead, namespaces are used extensively to provide modularity and prevent name collisions, so that all functions and variables belonging to a particular module are wrapped in the same namespace, whose name is derived from the name of the corresponding module in the host portion of the code.

### The simulator class

tledSimulator is the normal entry point for anyone wanting to use *NiftySim* as an FEM backend. A major motivation for the introduction of this class was the encapsulation of all simulation components except the model, and thus, the facilitation of the integration of *NiftySim* as an FE backend in C$++$ code, as was illustrated with the example in Fig. 3. Its most important member function, Simulate, contains the time stepping loop.

### The model class

The tledModel class is the in-memory representation of the simulation description, usable by the other components of *NiftySim*. Internally, it stores the XML description of the simulation as a Document Object Model (DOM) tree whose contents are accessible through member functions of tledModel.

A model can be defined recursively in XML through the notion of *sub-models*. Each sub-model is represented by its own tledModel instance whose management is done by tledSubModelManager.

### The mesh representation

The tledMesh class only provides basic information about the mesh, such as node positions and element connectivity; for more complicated topological queries, tledMesh Topology can be used. There is one instance of tledMesh accessible through the simulation’s model whose purpose is to hold all solid-element geometry in the simulation, even if a simulation contains multiple disjoint bodies, as is the case with many contact problems.

*NiftySim* provides its own mesh file format, which is based on an inline definition of meshes through a block of node positions and a block of element connectivities, in the simulation XML description, but it also supports reading of VTK unstructured grid files and the MSH^8^ ASCII file format. Further, it can output simulation results in VTK unstructured grid files (see section “Output”).

*NiftySim* also has some limited mesh manipulation capabilities, allowing it to apply affine transforms to meshes read from files and to assemble larger connected meshes from the meshes contained in sub-models. The sub-model manager performs this mesh merging operation incrementally by searching for nodes whose positions are less than a user-specified distance apart. Therefore, its use is recommended only on conforming meshes.

There are dedicated surface-mesh classes for holding membrane and shell elements (see section “tledShellSol verCPU”) and contact modelling (see section “Contact modelling”); all these classes are derived from tledSurface. The geometrical information necessary for shell and membrane computations is contained in a tledShellMesh instance that in turn depends on a solid mesh for the vertex positions. In cases where a solid body is wrapped in a membrane, the 2D mesh’s connectivity information is directly obtained from the solid mesh by extracting its surface facets. tledRigidContactSurface is used for the modelling of contacts with arbitrarily meshed rigid bodies and tledDeformableContactSurface holds the current-configuration surface for contact modelling purposes.

### The solver classes

The purpose of tledSolver and its sub-classes is the coordination of the time step calculations involved in completing the simulation: compilation of internal forces and external loads, imposition of BCs, and update of displacements.

#### tledSolverCPU

tledSolverCPU is the sequential C$++$ solver implementation of *NiftySim*. Precomputations of $\varvec{M}$, $\varvec{\partial h}$, etc., are performed in the class’s constructor. The main computational tasks in each time step are calculation of new internal nodal forces and calculation of new nodal displacements. The latter task is fully delegated to a dedicated CPU time-ODE solver class (described in section “Time integration”). The sequential loop by which the former calculation is carried out is summarised in the pseudo-code loop at the centre of Algorithm 1.

The element-level calculations are performed by element classes, each of which is derived from tledElement. Concrete classes are provided for the three solid-element types described in section “The basic TLED algorithm”. The element objects are managed by the solver object. Each element object also has an associated material object (of base class tledMaterial), which is responsible for the constitutive behaviour of the element and enables evaluation of stress, given the element deformation. The available constitutive models are described in section “Constitutive models” in Appendix. The task of computing BC values and body forces for a given time is performed by a *constraint manager* (described in section “Constraints”), but their accumulation and application is done by the solver. If applicable, a contact manager (tledContactManager) also resolves contacts between bodies in the model (see section “Contact modelling”).
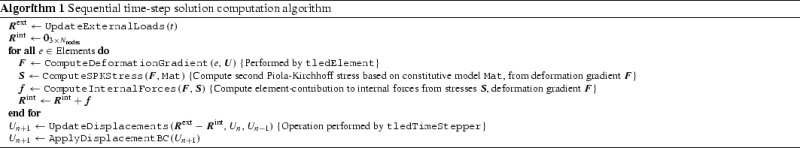


#### tledParallelSolverCPU

tledParallelSolverCPU is a parallel CPU solver based on Boost^9^ threads. It shares most of its code with tledSolverCPU. Its main distinguishing feature is that it splits the element array into blocks of equal size and assigns these sub-arrays to different threads. To avoid race conditions on the internal-forces buffer $\varvec{R}^\mathrm{int}$, every thread is associated with one intermediate force accumulation buffer, into which the internal forces of the elements in its sub-array are written. These temporary buffers are then summed up and the result is written to the global internal-force array.

#### tledSolverGPU

The nVidia CUDA solver implementation is called tledSol verGPU. All its precomputations are performed on the CPU with code resembling that of tledSolverCPU.

**Fig. 6:**
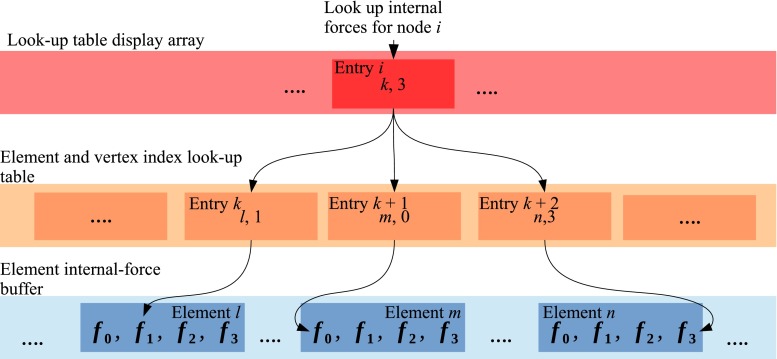
Layout of the buffer used for storage of internal forces on the GPU and illustration of their retrieval during computation of the effective loads

With most element types, only one kernel is required for the computation of the internal forces, which is invoked with one thread per-element. While conceptually there are few differences between that kernel and the loop body in Algorithm 1, the storage format for the element internal-forces is significantly different in that every element is assigned a float3 buffer of size $N_{\text {nodes/element}}$ in which only the forces computed by one thread for one element are held (Fig. 6). These forces are later retrieved in the displacement update stage. Thanks to this storage format, no inter-thread communication or atomic operations are required.

The second important solver kernel, the displacement update kernel, is invoked by the solver with one thread for every node. As is the case on the CPU, code associated with the solver is responsible for computation of the effective loads. The accumulation of the internal forces acting on a thread’s node is performed by querying two texture arrays, one display array of type int2 holding an offset and a range, and a second int2-array holding for every node the indices of the elements to which it belongs and its vertex index in those elements. Hence, these two arrays allow for a retrieval of all internal forces computed per element from the buffer that was filled by the internal-forces kernel. The look-up process is illustrated in Fig. 6. The external loads are computed on the CPU and passed as a global memory array to the kernel. The kernel is templated with respect to the tledTimeStepper sub-class used for displacement evolution, and the effective forces are next passed to the appropriate tledTimeStepper function via template polymorphism that in turn returns a predictor displacement value for the thread’s node. It is then checked if any of the node’s components are subject to constraints through a binary mask held in texture memory, with one entry for every component of every node. If the component is constrained, the corresponding value is retrieved from another texture array.

An example of the handling of contact constraints on GPUs is given in section “Contact modelling”.

#### tledSolverGPU_ROM

Reduced Order Modelling is implemented in the tledSol verGPU_ROM class, which follows a similar execution model to the basic GPU-enabled solver described in the previous section. In particular, computation of element nodal force contributions is identical to that in tledSolverGPU. The subsequent displacements update, however, is divided into a sequence of device and host computations: (i) effective nodal loads $\varvec{R}^\mathrm{eff}$ are assembled using a first kernel, launched over $N_{\text {nodes}}$ threads, then transferred to the host; (ii) the quantity $\varvec{\varPhi \hat{M}\varPhi ^{\text {T}}R}^\mathrm{eff}$ is computed and the resulting vector is transferred back to the device; and (iii) the final displacements $\varvec{U_{n+1}}$ are computed using a second kernel, also launched over $N_{\text {nodes}}$ threads. It is found to be more effective to perform step (ii) on the host side, as the small sizes of the involved vectors and matrices make GPU execution inefficient.

Matlab code for constructing the reduced basis from training data using proper orthogonal decomposition is also included in the *NiftySim* source code package.

#### tledShellSolverCPU

Similar to how tledSolverCPU is responsible for the spatial discretisation with solid elements on the CPU, the tledShellSolverCPU class performs the tasks of computing the mass of shell and membrane elements and their internal forces.

Element sets are implemented as classes templated with respect to the membrane element type, so as to allow for a mix of membrane/shell element types in the same simulation. These templated classes are derived from a common abstract class tledShellSolver::ElementSet that has a pure virtual function ComputeForces that is responsible for the computation of internal forces in one element set and receives a reference to the same buffer $\varvec{R}^\mathrm{int}$ used for accumulation of solid-element internal forces by tledSolverCPU. The contents of this function and its method of operation are largely analogous to the loop body of Algorithm 1, i.e. (i) the computation of strain/curvature measures is delegated to element classes derived from tledElementMembrane; (ii) a shell/membrane constitutive model object associated with the element set is used for computation of the stresses arising from the strains/curvatures; (iii) the element class converts the stresses to internal forces. Since the same force accumulation buffer is used as for solid elements, all BC and contact modelling operations can be performed by tledSolverCPU.

A class tledParallelShellSolverCPU exists to provide CPU parallelism. Its element set classes work by splitting their element arrays into equal parts that are assigned to different threads, very similar to how it is performed in tledParallelSolverCPU.

#### tledShellSolverGPU

tledShellSolverGPU is the CUDA implementation of tledShellSolverCPU. Its internal organisation and a large amount of administrative and precomputation code are shared with tledShellSolverCPU. As with its CPU counterpart, one design goal of this class was to reuse solid-element solver code for BCs, contact modelling, etc. The strategy for force accumulation employed by tledShellSolverGPU is largely identical to that of tledSolverGPU, i.e. forces are computed and stored element-wise, to be later retrieved by a dedicated kernel invoked with one thread per node using the same type of lookup tables. The aggregated forces are directly subtracted from the external loads before these are passed to the displacement update kernel of tledSolverGPU.

The internal-forces kernel is templated with respect to the constitutive model and element class, and the appropriate functions for computation of the deformation, stresses, and internal forces are called via template polymorphism.

### Time integration

The base class of all ODE solvers used for the time integration is tledTimeStepper. Two further abstract classes, tledTimeStepperCPU and tledTimeStepperGPU, exist to provide the CPU and GPU specific parts of the ODE solver API, respectively. Mathematically, two types of explicit time integration are supported: the central difference method and explicit Newmark integration (see section “The basic TLED algorithm”).

In order to maximise code reuse and consistency between the CPU and GPU implementations a design pattern based on templated decorators, which is used in several places in *NiftySim*, was employed. In this case, the CDM/EDM-specific but platform-independent parts of the implementation, e.g. getters for intermediate results such as velocity, are contained in two templated decorator classes, tledCentra lDifferenceTimeStepper and tledNewmarkTime Stepper. These decorators derive from a solver base class that is passed as a template argument, as follows
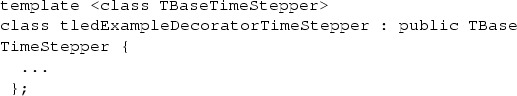


where TBaseTimeStepper is either tledTimeStep perCPU or tledTimeStepperGPU. These decorated CPU/GPU ODE solver base classes then serve as the parent class for the actual solver implementations, such as tledCentralDifferenceTimeStepperCPU.

The displacement evolution code of the GPU ODE solvers is implemented as a device function that is directly called by the displacement update kernel of the GPU solver. Unlike with the internal force computation, no precautions need to be taken to avoid race conditions, since the computation of the next displacement value of a given node only depends on its effective loads, and its current and previous time-step displacements.

### Constraints

Loads and boundary conditions are incorporated under the common heading of constraints. All constraint types are represented by a sub-class of tledConstraint, e.g. tledDispConstraint implements nonzero essential boundary conditions. A class called tledConstraint Manager is responsible for their management.

The constraint types accessible through the simulation XML description were originally aimed at an algorithmic generation of boundary condition definitions. Mostly, they are of a very basic type, such as displacement or force constraint, and require an explicit specification of the nodes directly affected by the constraint, thus making it difficult for humans to read and manually specify. More recently, we have added a method of geometric boundary specification that allows the user to specify the surface facets contained in a boundary through a combination of facet normal-orientation criteria and bounding volumes. The processing and conversion to node index lists of these descriptions is done in tledModel with the aid of the classes tledMeshSurface, that can extract surfaces of solid meshes and compute facet normals, and tledNodeRejector and its sub-classes that are used to filter nodes based on “is inside volume”-type criteria.

### Contact modelling

#### Contacts with analytically described surfaces

This feature enables the efficient simulation of contacts between soft tissue and geometries frequently encountered in medical settings. Examples of analytical contact-surface classes are tledContactCylinder and tledContact Plate. There is no common interface for analytical contact surfaces since these are very simple classes holding only a few parameters necessary to describe the surface, such as the radius, the axis and origin of the centre line in the case of the contact cylinder.

For performance reasons, the actual computations related to these contacts are performed by tledSolverGPU in the displacement update kernel. Algorithm 2 shows the computations performed to detect and simulate a contact between the deformable simulation geometry and a plate suitable for simulation of the breast compression in mammography. 
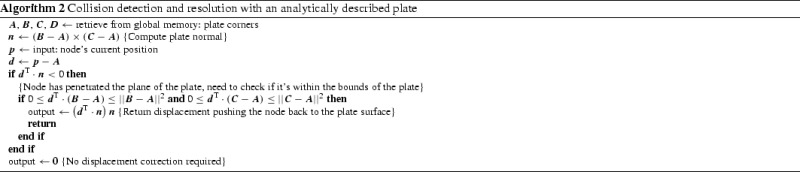
 No CPU equivalent exists for the analytical contact-surface feature.

#### Mesh-based contact modelling

A wide-range contacts can be modelled with the mesh-based code: contacts of multiple deformable bodies, deformable-body self-collisions, contacts between moving and static rigid bodies and deformable ones. A dedicated manager, tledUnstructuredContactManager, exists to manage the surface meshes used in the collision queries, the contact search bounding volumes, and the *contact solvers* that compute the collision response forces. Similar to how the constraint manager provides loads and boundary displacements to the solver for a given point in time, this manager provides member functions that can be called by the solver to get the forces arising from collisions for a given displacement configuration without needing any in-depth knowledge of the type of contacts simulated or the number of bodies involved in the contacts.

tledUnstructuredContactManager encapsulates one object holding the surface of the simulation geometry at the current time step, of the class tledDeformable ContactSurface. This data structure provides the facilities needed to construct a BVH for broad-phase contact search, the connectivity and surface-geometry information needed for the narrow-phase search and response-force computation. The BVH is a data structure that recursively partitions the geometry until every bounding volume (BV) only contains one surface primitive (e.g. a triangle). This partitioning is done such that when a BV is split, its children are only assigned geometric primitives that are connected.

The contact search is conducted in two phases: The broad phase operates only on the BVH and, in the case of deformable-body contacts, recursively checks sub-trees of the BVH containing geometry between which there is no topological connection, against each other. In this *pair-wise descent*, the geometry bounded by one BVH subtree is considered the master surface, the other is the slave.

The subsequent narrow-phase distinguishes between two types of contacts; mesh-intersections caused by slave nodes penetrating into master-surface facets and edges intersecting. The algorithm for the detection and correction of deformable-body intersection is summarised in pseudo-code, in Algorithm 3. 
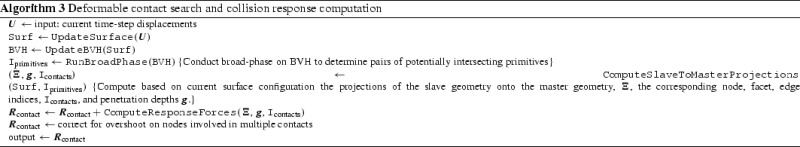


Conceptually, little changes with deformable and rigid body contact. The main difference is that each rigid contact surface is contained in its own data structure and has its own BVH. In the contact search, the entire deformable-body BVH is checked against the entire BVH of the rigid body. Further, contact-response forces are applied to the deformable body only.

In self-collision detection, the subtrees of the deformable-geometry BVH that need to be checked against each other are identified with the surface-cone method of Volino and Magnenat-Thalmann [31]. Otherwise, the algorithm is identical to Algorithm 3.

The template-based decorator design pattern described in section “Time integration” is used extensively to share code between the various mesh-based contact modelling pipelines. The mesh-based contact modelling is only available in the development branch of the project and not part of the stable releases, as of version 2.3.

### Output

#### Visualisation

Some basic visualisation capabilities are included in *NiftySim* ; these employ VTK for the rendering and window management. A custom render scene interactor, the *mesh sources*, which handle the conversion of *NiftySim* mesh objects and their attributes to VTK objects, and the source code for the creation of the render scene itself are contained in a separate library called libviz.

#### Mesh output

The same converters that are used in the visualisation module can be used to export the simulation mesh with the final displacement as an attribute in VTK’s vtkUnstructured Grid format, or vtkPolyData in the case of membrane meshes. This functionality can be invoked through the *NiftySim* front-end with the -export-mesh, -export- submesh, and -export-membrane switch for the export of all simulation geometry as one mesh, as individual submeshes, and surface meshes, respectively.

#### Displacement and internal force history

tledSimulator also encapsulates an instance of tled SolutionWriter which can record the time step displacements and internal forces. The displacements/forces are recorded in a Matlab parsable ASCII format at a frequency the user specifies through an attribute on the Output XML element that is used to request the output of a variable ($\varvec{F}$ or $\varvec{U}$).

## Research applications of NiftySim

In this section, we will look at a series of applications of *NiftySim* in published research. The majority of these examples illustrate the use of *NiftySim* for soft tissue simulations and exploit the speed of the GPU solver to run a large number of simulations with different parameters within a useful timeframe, e.g. to compute optimal material parameters for an image registration. However, in some cases *NiftySim* was also chosen for its features that go beyond TLED, such as its wide range of constitutive models or its contact modelling.

### Biomechanically guided prone-to-supine image registration of breast MRI using an estimated reference state

**Fig. 7:**
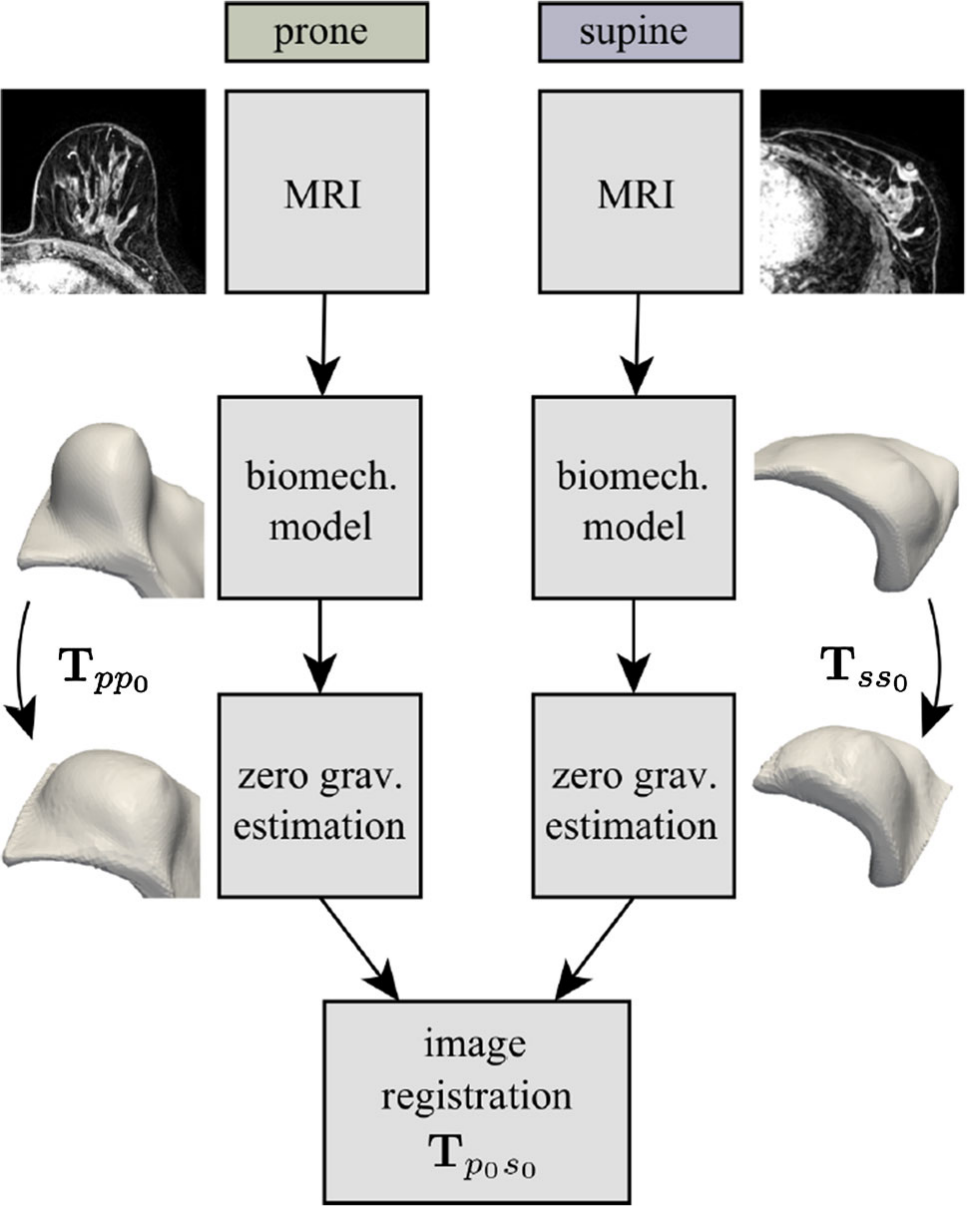
Overview of zero-gravity configuration estimation algorithm from Ref. [6]

This example application by Eiben et al. [6] aims to improve the results of registration of breast magnetic resonance images (MRI) from a prone to a supine patient position. The clinical motivation is that diagnostic images used in detecting breast cancer and the planning of its surgical removal are typically acquired with the patients lying on their stomach (prone). The interventions are performed with the patients lying on their back (supine) and may be guided with intra-operative imaging. Due to the softness of breast tissue, the deformation the breast undergoes between these two configurations is too large for standard image registration algorithms to cope with. For this reason, Eiben et al. proposed to estimate an artificial zero-gravity state for the pre-operative as well as the intra-operative images, in which correspondences between the two configurations can be established more easily, and subsequently refined to provide a starting position for standard B-spline nonrigid image registration. Figure 7 shows the algorithm as a diagram.

The implementation of this algorithm used *NiftySim* to simulate the unloading of the breast. To this end models comprising three neo-Hookean element sets with distinct parameters, taken from the literature, were constructed; corresponding to the pectoral muscle, the adipose tissue, and the fibro-glandular tissue. The reference state was obtained by using a gravity constraint on a mesh obtained from the loaded configurations and inverting the direction of gravity. This yields the reference configuration for a subsequent iterative refinement of the zero-gravity configuration. The refinement of the reference state is carried out by reloading the estimated zero-gravity mesh with the physical gravity direction and computing the difference between the loaded estimate and the configuration seen in the corresponding MR image. This difference is subsequently transformed back into the coordinate system of the reference configuration by means of a nodally averaged deformation gradient, and directly added to the vertex positions of the reference-configuration mesh:

26 $$\begin{aligned} \begin{aligned}&\varvec{\varDelta x_r} = \varvec{F}^{-1}\varvec{\varDelta x_l}\\&\varvec{x_r}^{(i+1)} = \varvec{x_r}^{(i)} + s\varvec{\varDelta x_r} \end{aligned} \end{aligned}$$

where the subscripts $l$ and $r$ are used to denote the loaded and the zero-gravity reference configurations, respectively, $\varvec{F}$ is the deformation gradient for the deformation from zero-gravity to loaded, $\varvec{x}_r$ denotes the node positions of the reference mesh, and $s \in ]0, 1[$ is a constant used to ensure convergence of the method.

Performing a validation based on landmarks in actual clinical data by tracking said landmarks from both the supine and prone configurations into the simulated reference configuration and measuring their distance, Eiben et al. obtained mean target registration errors (TREs) of 5.3–6.8 mm which is well below the clinically relevant threshold of 10 mm.

In their experiments, the algorithm required 19 simulations to converge both from the supine and prone configurations to the zero-gravity reference configuration. The simulations took an average 80 and 83 s on an nVidia GeForce GTX 580, respectively, with meshes with 10,455 and 10,741 nodes, respectively.

### Development of patient-specific biomechanical models for predicting large breast deformation

Han et al. [9] presented an algorithm for recovering suitable material parameters from MR images for the accurate modelling of breasts undergoing large deformation, such as in the previously discussed prone-to-supine registration. The algorithm was used to estimate material parameters for up to four different types of tissue within a model: fat, fibro-glandular, muscle, and tumour tissue. The inputs were: a segmented image of the initial (subsequently denoted by $A$) and final configurations (called $B$), and a set of initial guesses for the material parameters that were obtained from the literature.

The algorithm was implemented with the unmodified stand-alone executable of *NiftySim*. It made heavy use of the element set concept, and if the experimental setup demanded it, *NiftySim* ’s contact modelling features. A pseudo-code description of the algorithm is given in Algorithm 4. 
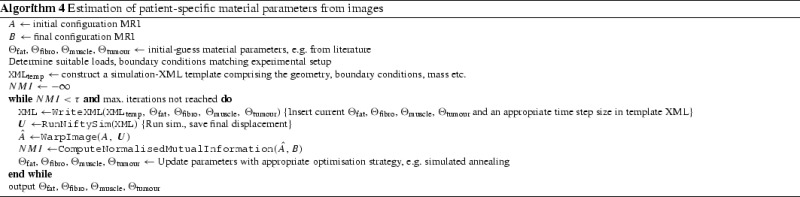


This iterative optimisation process was effectively enabled by the speed advantages of *NiftySim* ’s GPU-enabled solver over established commercial packages: individual simulations took 19 s to complete with *NiftySim*, compared with 104 min with ABAQUS standard and 312 min with ABAQUS explicit on an Intel dual-core 3.4 GHz CPU with a GeForce GTX 285 GPU. They also ascertained that *NiftySim* ’s solutions are consistent with those obtained with the slower commercial packages.

### Modelling prostate motion for data fusion during image-guided interventions

**Fig. 8:**
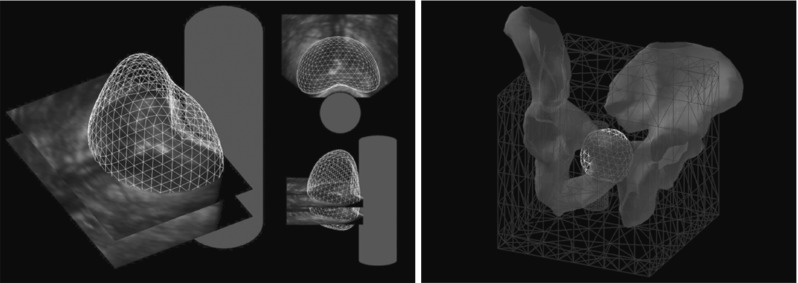
*Left* Parallel TRUS images and the corresponding extracted prostate gland surface mesh, and a simplified TRUS probe balloon indicating the position of the probe during acquisition. *Right* Example of a simulation mesh used by Hu et al. with the pelvis used for defining the essential boundary conditions

Hu et al. [13] described an approach to registering intra-operative transrectal ultrasound (TRUS) images with, for example, pre-operative MR images, for guidance of prostate biopsy procedures. Statistical Motion Models (SMMs), constructed pre-operatively, are aligned to the intra-operative TRUS images, which process may be performed in real-time. In the process, they define a dense deformation field throughout the image volume, which may be used as a high-quality initialiser for a fine registration with an intensity-based method. The SMMs are constructed off-line from the results of a series of FE simulations, carefully designed to ensure the parameter space of the problem is adequately sampled. An example of a TRUS image with an extracted prostate mesh and a simplified TRUS probe can be seen in Fig. 8.

Their FEM models consisted of a prostate gland embedded in a rectangular block with a hole representing the rectum. *NiftySim* ’s tledContactUSProbe class was used to simulate the ultrasound probe’s motion and interaction with the tissue. The FEM models comprised four element sets corresponding to the prostate inner and outer gland, rectal wall, and other surrounding tissue. Further, they used a generic pelvis model with random rotation, translation, and scaling parameters to impose a homogeneous displacement constraint on the model (Fig. 8). An outline of the implementation of the SMM generating algorithm can be found in Algorithm 5 
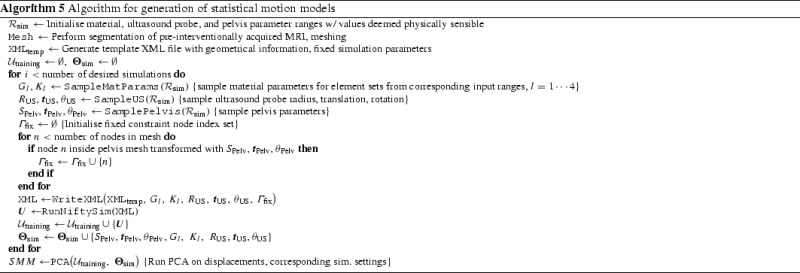


Using *NiftySim* ’s GPU-enabled solver, a full training set of 500 simulations were completed in an average of 140 min and with minimal user intervention, rendering the process amenable to clinical use. By comparison, comparable (individual) simulations using Ansys take between 10 and 30 min. Using these statistical models Hu et al. were able to obtain TREs of $<$3 mm, which is both below the clinically relevant threshold of 4.92 mm and the TREs obtained with elastic registration that they identified as the primary competing method.

### MRI to X-ray mammography intensity-based registration with simultaneous optimisation of pose and biomechanical transformation parameters

Mertzanidou et al. [20] developed a method for registering 3D MR images to 2D X-ray mammograms. The problem is particularly challenging as the X-ray images are acquired with the breast being compressed between two plates. The MRIs are also used diagnostically and for surgical planning, and are acquired with the women lying prone with their breasts pendulous. The algorithm aims to simulate the compression on a mesh generated from an MRI, using the resulting displacement field to warp the MRI, and generate a simulated X-ray of the compressed MRI via ray-casting. Finally, the simulated X-ray is repeatedly compared with the actual X-ray mammogram, thus at convergence, providing correspondences between the two images of the breast, as assessed by the normalised cross- correlation (NCC) metric. Simulations were performed using *NiftySim* and making use of a transversely isotropic neo-Hookean constitutive model for the breast tissue with a fixed Young’s modulus. The other material parameters were optimised as part of the registration procedure, in a manner similar to that proposed by Han et al. [9]. A pseudo-code summary of the algorithm is given in Algorithm 6.

The algorithm was implemented in a dedicated application using *NiftySim* ’s GPU solver as a backend to save the time required to reload the simulation model, by substituting material parameters, using tledSolverGPU’s UpdateMaterialParams function, and the displacement settings of the tledContactPlate contact surfaces in every iteration of the hill-climbing optimisation. However, it could be implemented using the @niftysim@ stand-alone application without making any functional sacrifices. Further, computational costs can be significantly reduced by performing the warping on-the-fly as part of the raycasting process.
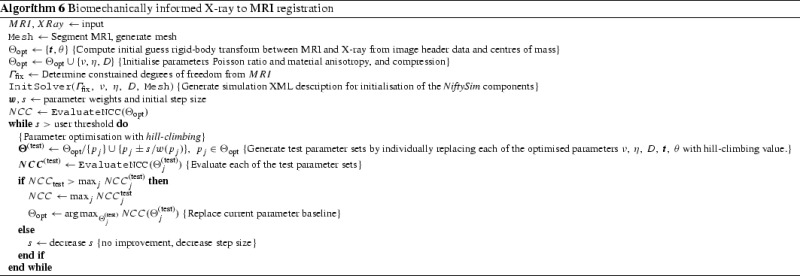
 The NCC evaluation function is given in Algorithm 7. 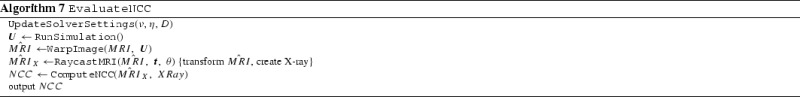


The use of *NiftySim* ’s GPU solver allowed Mertzanidou et al. to run approximately 420 simulations in one registration, taking about 2 hours in total.

They obtained TREs of $11.6\,\pm \,3.8$ and $11\,\pm \,5.4$ mm for the registration of the MRI to the cranio-caudal and the medio-lateral oblique X-ray, respectively.

The algorithm presented by Mertzanidou et al. aims to solve one of the most difficult problems commonly encountered in medical image registration, but for the purposes of this paper, it is also notable for its use of some of *NiftySim* ’s newer features. In addition to the above algorithm, that uses a frictionless analytical model for the contact plates and a homogeneous solid-element model, they also performed a sensitivity analysis to assess the impact of a more sophisticated model including a membrane representing the patient’s skin, and friction between the contact plates and the breast surface. The incorporation of friction requires using the mesh-based contact model, and the creation of a surface mesh for the contact plates. The “skinning” of the mesh with a neo-Hookean membrane as done by Mertzanidou et al. can be achieved with the following lines of XML code: 
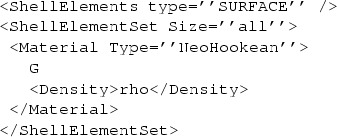
 where G and rho are a suitable shear modulus and mass density, respectively.

**Fig. 9:**
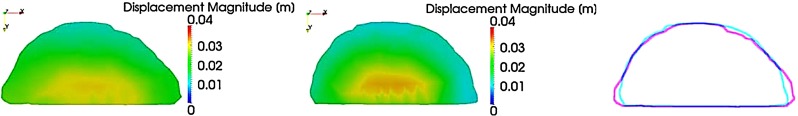
Qualitative comparison of simulations without (*left*) and with (*centre*) a skin-simulating membrane by means of final configuration cross sections and their contours (*right*)

With a friction coefficient of $\mu = 0.3$ and a skin shear modulus twice that used for the breast solid mesh, they observed the following effects when compared to the frictionless homogeneous model: 4.89 mm mean difference in nodal 3D displacement, and 4.36 mm mean difference in axial displacement. Figure 9 shows a qualitative assessment of the effects of the skinning performed by Mertzanidou et al. in which they looked at cross sections through the simulation final configurations.

## Discussion and conclusions

The *NiftySim* toolkit has been designed to enable efficient integration of simulation technology into applications in medical image computing and computer-assisted interventions. This integration is facilitated by both a command line program capable of executing simulations in a stand-alone fashion, and a library which enables simple embedding of the simulation code in third-party software. High computational performance is achieved by employing a highly data-parallel FE algorithm and executing on massively parallel graphics processing units. The underlying formulation is valid for fully nonlinear problems, making it suitable for simulating materially nonlinear soft tissues undergoing large deformations. Moreover, the codebase is relatively small and minimally dependent on third-party libraries, allowing fast and easy compilation on a range of platforms, and an uncomplicated integration in client code. A series of example applications from recently published work was used to demonstrate the toolkit’s utility.

## Appendix: Constitutive models

Constitutive models in *NiftySim* are defined in terms of scalar valued strain energy density functions $\varPsi$. From these, the $2$nd Piola–Kirchhoff stress $\varvec{S}$ may be computed using

27 $$\begin{aligned} \varvec{S} = \frac{\partial \varPsi }{\partial \varvec{E}} = 2 \frac{\partial \varPsi }{\partial \varvec{C}}. \end{aligned}$$

in which we have introduced the right Cauchy–Green deformation $\varvec{C} := \varvec{F}^\mathrm{T} \varvec{F}$.

For isotropic elastic models, $\varPsi$ is a function of deformation only: $\varPsi = \varPsi (\varvec{C})$. We employ strain energy functions with separated isochoric (volume-preserving) and volumetric components [11], thus:

28 $$\begin{aligned} \varPsi (\varvec{C}) \!=\! \varPsi ^\mathrm{iso} (\bar{\varvec{C}}) \!+\! \varPsi ^\mathrm{vol} (J) \!=\! \varPsi ^\mathrm{iso} (\bar{I}_1, \bar{I}_2) \!+\! \varPsi ^\mathrm{vol} (J), \end{aligned}$$

where $J := \det \varvec{F}$ is the Jacobian determinant, $\bar{\varvec{C}} = J^{-2/3} \varvec{C}$ is the modified right Cauchy-Green deformation tensor, and $\bar{I}_1 = \mathrm{tr} \bar{\varvec{C}}$ and $\bar{I}_2 = \big [ ( \mathrm{tr} \bar{\varvec{C}} )^2 - \mathrm{tr} ( \bar{\varvec{C}}^2 ) \big ]/2$ are invariants of $\bar{\varvec{C}}$.

Transversely isotropic models, characterised by a single “preferred” direction $\varvec{a_0}$ and symmetrical properties orthogonal to this, are formed through the addition of terms dependent on the pseudo-invariant $\bar{I}_4 = \varvec{a_0} \cdot \bar{\varvec{C}} \varvec{a_0}$.^10^ In this case $\varPsi$ becomes

29 $$\begin{aligned} \varPsi (\varvec{C,a_0}) = \varPsi ^\mathrm{iso}(\bar{I}_1,\bar{I}_2,\bar{I}_4) + \varPsi ^\mathrm{vol}(J). \end{aligned}$$

Finally, visco-hyperelastic models may be formed by augmenting elastic strain energy functions with time-dependent relaxation functions $\alpha (t)$ and integrating over the history of the loading:

30 $$\begin{aligned} \hat{\varPsi }(\varPsi ,t) = \int _0^t \alpha (t-s) \frac{\partial \varPsi }{\partial s} \mathrm{d}s. \end{aligned}$$

*NiftySim* visco-hyperelastic constitutive models use the common Prony series form of relaxation function:

31 $$\begin{aligned} \alpha (t) = \alpha _{\infty } + \sum _{i=1}^N \alpha _i \mathrm{e}^{-t/\tau _i}, \end{aligned}$$

where $\alpha _{\infty }$, $\alpha _i$, and $\tau _i$ are positive real constants.

### Hyperelastic models

The following hyperelastic strain energy functions are currently available:

#### Neo-Hookean

32 $$\begin{aligned} \varPsi _\mathrm{NH} = \frac{\mu }{2} \left( \bar{I}_1 - 3 \right) + \frac{\kappa }{2} \left( J - 1 \right) ^2, \end{aligned}$$

where $\mu$ and $\kappa$ are the shear and bulk moduli, respectively.

#### Polynomial

33 $$\begin{aligned} \varPsi _\mathrm{PY} = \sum _{i+j=1}^{N} C_{ij} \left( \bar{I}_1 - 3 \right) ^i \left( \bar{I}_2 - 3 \right) ^j + \sum _{i=1}^{N} \frac{1}{D_i} \left( J - 1 \right) ^{2i},\nonumber \\ \end{aligned}$$

where $C_{ij}$ and $D_i$ are material parameters (related to the initial shear and bulk moduli as $\mu = 2(C_{10} + C_{01})$ and $\kappa = 2/D_1$), and $N=2$.

#### Arruda–Boyce

34 $$\begin{aligned} \varPsi _\mathrm{AB} = \mu \sum _{i=1}^{5} \frac{C_{i}}{\lambda _m^{2i-2}} \left( \bar{I}_1^i - 3^i \right) + \frac{\kappa }{2} \left( \frac{J^2 - 1}{2} -\ln J \right) ,\nonumber \\ \end{aligned}$$

where $\mu$ and $\kappa$ are the initial shear and bulk moduli, respectively, $\lambda _m$ is the locking stretch, and $C_{i}, (i=1,\ldots ,5)$ are constants: $C_1=1/2$, $C_2=1/20$, $C_3=11/1050$, $C_4=19/7{,}000, C_5=519/673{,}750$.

#### Transversely isotropic

35 $$\begin{aligned} \varPsi _\mathrm{TI} = \frac{\mu }{2}\left( \bar{I}_1 - 3 \right) + \frac{\eta }{2}\left( \bar{I}_4 - 1 \right) ^2 + \frac{\kappa }{2} \left( J - 1 \right) ^2, \end{aligned}$$

where $\eta$ is a material parameter (units of Pa) controlling the additional stiffness in this direction.

### Visco-hyperelastic models

Viscoelastic versions of the neo-Hookean and transversely isotropic models are currently available. See [28] for a description of the constitutive update procedure for visco-hyperelastic materials.

## Footnotes

1 Simulation Open Framework Architecture, available from http://www.sofa-framework.org.

2 Abaqus FEA is a product of Dassault Systèmes, http://www.3ds.com/products-services/simulia/portfolio/abaqus/.

3
*NiftySim* supports CMake versions $\ge \! 2.8$ obtainable from http://www.cmake.org.

4
http://sourceforge.net/projects/niftysim/.

5 Doxygen is a tool for the extraction of inline API documentation, available from http://www.doxygen.org.

6 Visualisation Toolkit: http://www.vtk.org.

7
http://www.itk.org.

8 MSH is the file format of the gmsh mesher available from http://www.geuz.org/gmsh.

9 Boost is an open-source library available from http://www.boost.org.
